# DIAgui: a Shiny application to process the output from DIA-NN

**DOI:** 10.1093/bioadv/vbae001

**Published:** 2024-01-13

**Authors:** Marc-Antoine Gerault, Luc Camoin, Samuel Granjeaud

**Affiliations:** Aix-Marseille University, INSERM, CNRS, Institut Paoli-Calmettes, CRCM, Marseille Protéomique, Marseille F-13009, France; Centrale Marseille School, Marseille F-13013, France; Aix-Marseille University, INSERM, CNRS, Institut Paoli-Calmettes, CRCM, Marseille Protéomique, Marseille F-13009, France; Aix-Marseille University, INSERM, CNRS, Institut Paoli-Calmettes, CRCM, Marseille Protéomique, Marseille F-13009, France

## Abstract

**Summary:**

DIAgui is an R package to simplify the processing of the report file from the DIA-NN software thanks to a Shiny application. It returns the quantification of either the precursors, the peptides, the proteins, or the genes thanks to the MaxLFQ algorithm. In addition, the latest version provides the Top3 and iBAQ quantification and the number of peptides used for the quantification. In the end, DIAgui produces ready-to-interpret files from the results of DIA mass spectrometry analysis and provides visualization and statistical tools that can be used in a user-friendly way.

**Availability and implementation:**

Code and documentation are available on GitHub at https://github.com/marseille-proteomique/DIAgui. The package is written in R and also uses C++ code. A vignette shows its use in an R command line workflow.

## 1 Introduction

Data-independent acquisition (DIA) proteomics is a recently developed global proteomics strategy based on mass spectrometry (MS). It is increasingly used in various proteomics studies as it offers broad protein coverage, high reproducibility, and accuracy (Hu *et al.* 2016, Krasny and Huang 2021). In a DIA acquisition, precursor ions are isolated within pre-defined isolation windows and then fragmented together, in contrast to conventional data-dependent acquisition (DDA) where they are isolated for a specific m/z. All fragmented ions in each window are then analyzed by a high-resolution mass spectrometer. The analysis of these much more complex MS2 spectra is now made easy thanks to artificial intelligence. Many software tools are now available, such as Spectronaut, DIA-NN, or Skyline. In a recent benchmark (Gotti *et al.* 2021), DIA-NN was found to be one of the most efficient. As it is also free, DIA-NN is increasingly used in the proteomics community. However, to fully use DIA-NN, its author strongly advises filtering the DIA-NN output using R and then applying the MaxLFQ algorithm to obtain a better quantification (Demichev *et al.* 2020). Furthermore, DIA-NN does not offer absolute quantification like the Top3 (Grossmann *et al.* 2010) or iBAQ (Brönstrup 2004) methods as in the state-of-the-art DDA software MaxQuant for example. In order to automate post-processing with R and give the possibility to compute absolute quantification from DIA-NN output, we propose DIAgui an R package (based on diann R package) that provides a *user-friendly* interface. Thanks to DIAgui, users can process DIA-NN results without knowing R and obtain more complete and interpretable results.

## 2 Description

DIAgui is applicable to all types of proteomic experiments as long as the data were processed through DIA-NN. Only the ‘report.tsv’ file is required. DIAgui contains two main functions: *report_process* which analyzes the report file with a single R command and *runDIAgui* which launches the Shiny application to process the report file in an interactive way.

**Figure 1.**
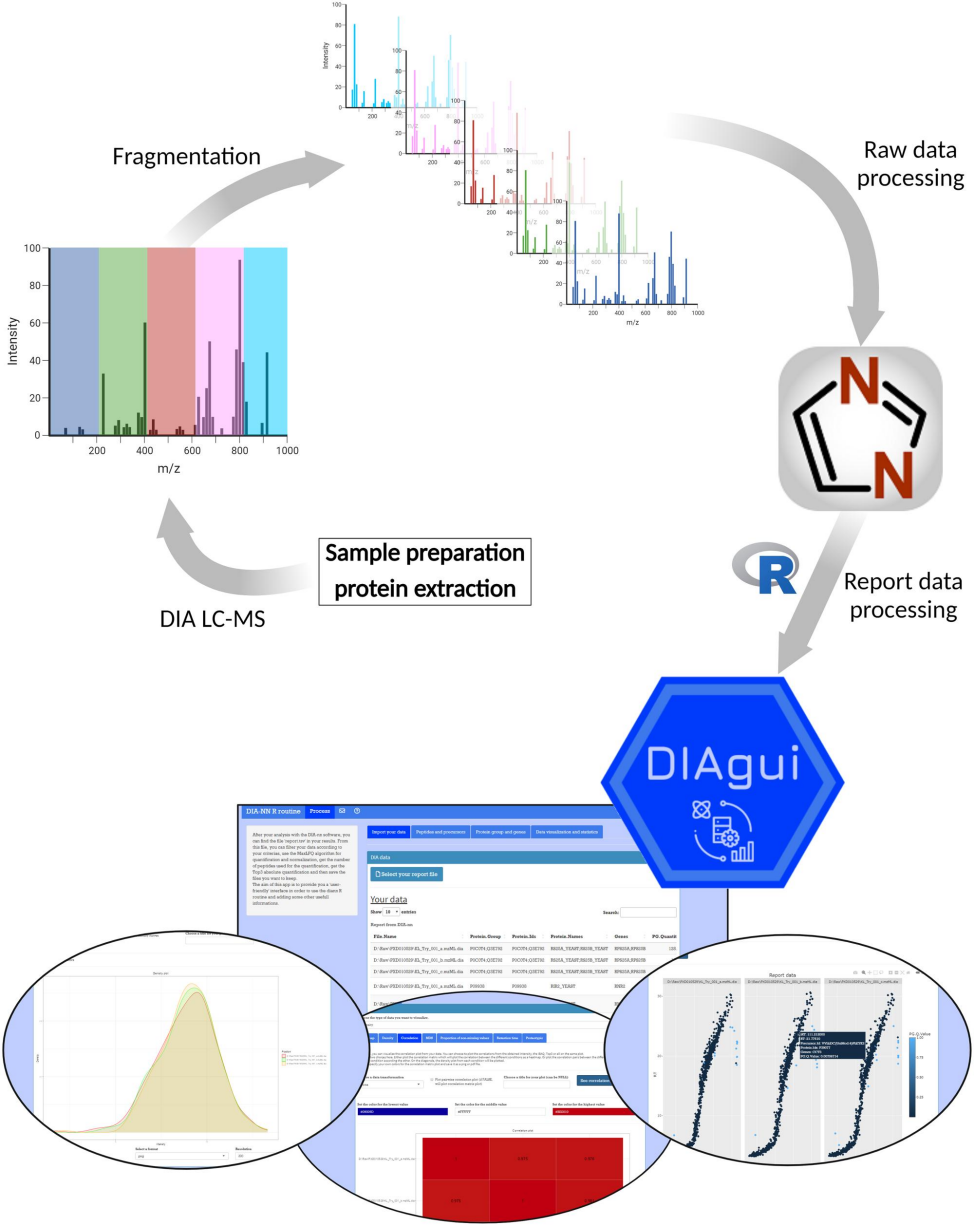
Workflow of DIAgui. After data independent analysis mass spectrometry (DIA-MS) of any protein sample, the raw data are analyzed with the software DIA-NN. Then using our R package DIAgui, the user can analyze the output from DIA-NN in a user-friendly way using our Shiny application accessible through DIAgui.

## 3 The Shiny application

The DIAgui app is divided into four main tabs. The first one allows the user to import the report file (usually named ‘report.tsv’) resulting from DIA-NN. When the data are loaded, the user can change the name of the LC-MS files which are by default the path of the raw files used. The next two tabs allow the extraction of datasets at different levels: precursors, peptides, proteins group, or genes. For each extraction, the user can filter precursors based on q-values, keep only proteotypic peptides and remove modified peptides. Each extraction can be downloaded in txt, CSV or Excel format. To extract the protein group dataset, the MaxLFQ algorithm (Cox *et al.* 2014) will be used with either the method from diann R package (https://github.com/vdemichev/diann-rpackage) (Demichev *et al.* 2020) or the iq R package method (Pham *et al.* 2020). The iq package code is much faster, but the results are totally equivalent. DIAgui can also calculate Top3 (Grossmann *et al.* 2010) and iBAQ (Brönstrup 2004) quantification. For iBAQ calculation, either the FASTA file used during the DIA-NN step must be loaded or the seqinr R package (Charif and Lobry 2007) must be used to query the SwissProt database (SwissProt Database 2008). Using a FASTA file is much faster because DIAgui performs no query to SwissProt. For precursors, peptides, or genes quantification, DIAgui is based on the *diann_matrix* function of the diann R package, meaning that the MaxLFQ algorithm is not used. In DIAgui, we improved this function by adding the Top3 quantification with the option to take the sum or the max of the intensities of the same ID. Taking the sum of these intensities is useful to obtain Top3 and iBAQ absolute quantifications, but it should be noted that this calculation uses the unnormalized raw intensity. Moreover, DIAgui reports the number of peptides used for the quantification, which is important to assess the quality of the quantification. Important additions to the quantification are summarized in the supplementary table. The last tab of DIAgui allows exploring the extracted dataset directly and easily. The user can visualize the data using an interactive heatmap, a density plot, correlation plot, and a MDS plot, or assess the proportion of non-missing values and much more. The user can also perform missing value imputation and statistical comparison between chosen groups. This exploration is offered for data filtered using DIAgui or initially uploaded data.

Finally, DIAgui can also help the user to study the impact of the m/z window selection of their DIA analysis. Using the *get_bestwind* function or the ‘Check other reports’ tab under the ‘Data visualization and statistics’ tab from the app, the user can see what the distribution of the precursors would be as a function of a fixed number of windows or a fixed window size, based on the report-Lib file output from DIA-NN. In addition, the user can allow a fixed overlap between windows. This feature could facilitate the development of DIA methods with variable windows.

All the features of DIAgui are presented in a video tutorial accessible by clicking the question mark icon or via https://youtu.be/vfvh15Q93eU.

## 4 Conclusion

DIAgui is freely available at https://github.com/marseille-proteomique/DIAgui. The user only needs to install R and follow the R package installation procedure. No R code skills are required. As DIA-NN is increasingly used in quantitative proteomics based on DIA acquisition, our *user-friendly* interface will allow the proteomics community to debride and accelerate the processing of their DIA analyses and research.

## Supplementary Material

vbae001_Supplementary_Data

## References

[vbae001-B1] Brönstrup M. Absolute quantification strategies in proteomics based on mass spectrometry. Expert Rev Proteomics 2004;1:503–12. 15966845 10.1586/14789450.1.4.503

[vbae001-B2] Charif D, Lobry JR. SeqinR 1.0-2: A contributed package to the R project for statistical computing devoted to biological sequences retrieval and analysis. In: Bastolla U, Porto M, Roman HE, Vendruscolo M (eds), *Structural Approaches to Sequence Evolution: Molecules, Networks, Populations*. Berlin, Heidelberg: Springer Berlin Heidelberg, 2007, 207–32.

[vbae001-B3] Cox J , Hein MY, Luber CA et al Accurate proteome-wide label-free quantification by delayed normalization and maximal peptide ratio extraction, termed MaxLFQ. Mol Cell Proteomics, 2014;13:2513–26. 24942700 10.1074/mcp.M113.031591 PMC4159666

[vbae001-B4] Demichev V , Messner CB, Vernardis SI et al DIA-NN: neural networks and interference correction enable deep proteome coverage in high throughput. Nat Methods 2020;17:41–4. 31768060 10.1038/s41592-019-0638-x PMC6949130

[vbae001-B5] Gotti C , Roux-Dalvai F, Joly-Beauparlant C et al Extensive and accurate benchmarking of DIA acquisition methods and software tools using a complex proteomic standard. J Proteome Res 2021;20:4801–14. 34472865 10.1021/acs.jproteome.1c00490

[vbae001-B6] Grossmann J , Roschitzki B, Panse C et al Implementation and evaluation of relative and absolute quantification in shotgun proteomics with label-free methods. J Proteomics 2010;73:1740–6. 20576481 10.1016/j.jprot.2010.05.011

[vbae001-B7] Hu A , Noble WS, Wolf-Yadlin A et al Technical advances in proteomics: new developments in data-independent acquisition. F1000Res 2016;5(F1000 Faculty Rev):419. 10.12688/f1000research.7042.1 PMC4821292 27092249

[vbae001-B8] Krasny L , Huang PH. Data-independent acquisition mass spectrometry (DIA-MS) for proteomic applications in oncology. Mol Omics 2021;17:29–42. 33034323 10.1039/d0mo00072h

[vbae001-B9] Pham TV , Henneman AA, Jimenez CR et al iq: an R package to estimate relative protein abundances from ion quantification in DIA-MS-based proteomics. Bioinformatics 2020;36:2611–3. 31909781 10.1093/bioinformatics/btz961 PMC7178409

[vbae001-B10] SwissProt Database. *Encyclopedia of Genetics, Genomics, Proteomics and Informatics, 1906-1906*. Dordrecht: Springer Netherlands, 2008. DOI: 10.1002/047001153X.

